# EVALUATION OF FUNCTIONAL CHARACTERISTICS IN PATIENTS WITH KNEE OSTEOARTHRITIS

**DOI:** 10.1590/1413-785220172506144577

**Published:** 2017

**Authors:** SERKAN BAKIRHAN, OZGUR BOZAN, BAYRAM UNVER, VASFI KARATOSUN

**Affiliations:** 1. Departments of Physical Therapy and Rehabilitation, Lefke, Faculty of Health Sciences, European University of Lefke, TRNC Mersin 10, Turkey.; 2. Private Clinician, Izmir, Turkey.; 3. School of Physiotherapy , Dokuz Eylül University, Izmir, Turkey.; 4. Department of Orthopedics, School of Medicine, Dokuz Eylül University, Izmir, Turkey.

**Keywords:** Osteoarthritis, Knee, Disability evaluation, Body weight, Osteoartrite, Joelho, Avaliação da deficiência, Peso corporal.

## Abstract

**Objective:**

This study evaluated the physical and functional characteristics of Turkish patients with knee osteoarthritis and how this disease affects their physical and functional status.

**Methods:**

This study included 320 patients, who were evaluated to assess body mass index (BMI) and Hospital for Special Surgery (HSS) score in terms of age, sex and functional characteristics.

**Results:**

Mean patient age was 66.92±8.89 years and mean BMI was 31.02±5.20 kg/m^2^. Mean patient HSS score was 58.70±11.08. According to their sit-to-stand test results, 33% of the patients (n=104) were found to be independent. There was a significant relationship between BMI and functional activity score (p<0.05).

**Conclusions:**

The majority of the patients in our study were female and obese, and had low functionality levels. Function in patients with OA is restricted as a result of excess weight, so preventive measures can help Turkish patients with OA maintain their ideal weight. Furthermore, patient education can be help this population acquire the habit of regular exercise in order to reduce pain and improve their physical activity and quality of life. **Level of Evidence IV, Case Series.**

## RESUMO

**Objetivo:**

Este estudo avaliou as características físicas e funcionais dos pacientes turcos com osteoartrite e como essa doença afeta seu estado físico e funcional.

**Métodos:**

O estudo incluiu 320 pacientes que foram avaliados quanto ao índice de massa corporal (IMC) e quanto ao escore Hospital for Special Surgery (HSS), em termos de idade, sexo e características funcionais.

**Resultados:**

A média de idade dos pacientes foi 66,92 *±* 8,89 anos e a média do IMC foi 31,02 *±* 5,20 kg/m^2^. A média do escore HSS dos pacientes foi 58,70 *±* 11,08. De acordo com os resultados do teste sentar/levantar, observou-se que 33% dos pacientes (n = 104) eram independentes. Houve relação significativa entre IMC e escore de atividade funcional (p < 0,05).

**Conclusões:**

A maioria dos pacientes em nosso estudo era do sexo feminino e obesos e tinham níveis baixos de funcionalidade. A função dos pacientes com OA foi restrita em decorrência do excesso de peso, de modo que as medidas preventivas podem auxiliar os pacientes turcos a manter o peso ideal. Além disso, a educação dos pacientes pode ajudar essa população a adquirir o hábito de exercícios regulares para reduzir a dor e melhorar a atividade física e a qualidade de vida. **Nível de Evidência IV, Série de Casos.**

## INTRODUCTION

Osteoarthritis (OA) is the most prevalent chronic rheumatic disease, and is the leading cause of pain and disability in most countries worldwide. Several epidemiologic studies have investigated risk factors for knee OA, finding a consistent association between the incidence or progression of knee OA and age, obesity, weight change, sex, history of knee injury, occupational physical demands, physical activity, lifestyle and geographic regions.^1^ The literature contains reports that physical characteristics, quality of life, pain, joint motion limitation, and functional activities of patients with knee OA are affected at different levels.^2^ ^,^ ^3^

The prevalence of OA varies in different geographic regions.^1^ Activities such as sitting on the ground, kneeling, sitting cross-legged, squatting and performing the *salaat* (a form of Islamic prayer) are common in Asian, Far Eastern, and Middle East cultures. During these activities which require high knee flexion, OA process can be triggered because of the increased pressure applied to the knee.^4^ Frequent repetition of these activities, which are an important part of daily life, leads to an increased incidence of OA in these societies. As in Far Eastern and Middle Eastern countries, the incidence of OA in Turkish population increases each year due to risk factors resulting from similar activities frequently performed by people in their daily lives, which, in turn, leads to significant limitations on their functional activities. It is estimated that 4% of the elderly Brazilian population has OA, with the knee being the second joint most affected by this disease, in 37% of cases.^5^ In a study conducted on the prevalence of OA in Turkey, the prevalence of symptomatic knee OA in the population over 50 years of age was determined to be 14.8%.^6^ Several studies have investigated the incidence and functional and physical impacts of OA in communities with life styles similar to Turkey,^7^ but no studies have investigated the functional and physical effects of OA on this population. The purpose of this study is to evaluate the physical and functional characteristics of osteoarthritis patients and how OA affects the physical and functional status of patients with knee OA in Turkish society.

## MATERIALS AND METHODS

A total of 320 patients (63 men and 257 women, mean age 66 years; range 40-87) with knee OA were included in the study. These patients were divided into 4 groups according to age: 40-59, 60-69, 70-79 and 80-89 years.

Body Mass Index (BMI) was defined as weight in kilograms divided by the square of patient’s height in meters. Patients were stratified by obesity status into 4 groups according to their BMI values: <25 kg/m² (underweight), 25-29.9 kg/m² (overweight), 30-39.9 kg/m² (obese), and ≥40 kg/m² (morbidly obese).

Physical knee function was evaluated in all patients using the Hospital for Special Surgery (HSS) knee score criteria, which is based on a total of 100 points. The score is divided into the following categories: lack of pain (30 points); function (22 points); range of motion (18 points); muscle strength (10 points); flexion deformity (10 points); and lack of instability (10 points).^3^ ^,^ ^8^ Active range of knee flexion was measured with a universal goniometer.^3^ Extensor mechanism function was evaluated at the same time using the Sit-to-Stand (STS) test.^9^ Patients were asked to rise from a 40-cm-high chair while keeping their arms folded across their chest.^10^ Quadriceps femoris (QF) muscle strength was assessed via the manual muscle testing method while the patient was in the sitting position, and a score ranging from 0 to 5 was assigned.^3^

SPSS 22.0 software was used for statistical evaluation of the data. Data were presented as mean and standard deviation. The one-way ANOVA test was used to compare variables in the groups. Results in which p<0.05 were considered significant. 

Our study is a retrospective study. The data were obtained by screening patient files. Therefore, ethic committee approval and patients’ consent were not obtained.

## RESULTS

This present study examined risk factors for knee OA among 320 Turkish people who ranged in age from 40 to 87 years. The majority of the patients were female (n=80%). (Table 1) All our patients had radiographic severity grade 4 OA on the Kellgren and Lawrence (KL) scale. 

**Table 1:** Demographic characteristics of the patients with knee osteoarthritis.

Age (year)	66.92±8.89 (40-87)
Sex (male/female)	63 M, 257 F
Weight (kg)	77.49±12.63 (50-117)
Height (cm)	158.28±7.36 (142-180)
BMI (kg/cm^2^)	31.02±5.20 (17.28-47.84)

BMI: Body Mass Index.

**Figure 1:**
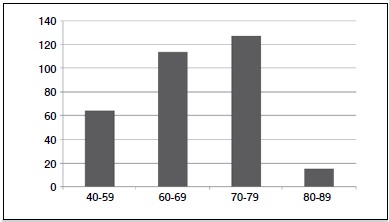
Patient age distribution.

The patients were classified with respect to age; 64 patients were 40-59 years old, 114 patients were 60-69 years old, 127 patients were 70-79 years old and 15 patients were 80-89 years old. (Figure 1)

**Figure 2:**
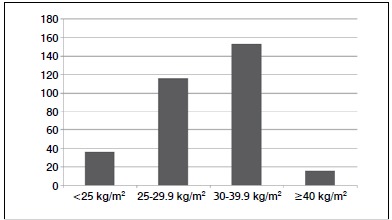
Patient BMI distribution.

BMI in the study population varied from 17.28 kg/m^2^ to 47.84 kg/m^2^, with a mean of 31.02 kg/m^2^. (Table 1) The study population was classified according to BMI as follows; underweight, 36/320 (11%); overweight, 116/320 (36%); obese, 153/320 (48%); morbidly obese, 15/320 (5%). (Figure 2)

The mean HSS score (0-100) was 58.70±11.08 (range 22-89). The mean HSS pain score was 10.95±7.10 (range 0-30), and the mean HSS functional activity score was 10.36±3.69 (range 4-22). The mean degree of active knee flexion was 100.36±16.45 (range 35-136). The transfer activity score was 2.46±1.09 (range 2-5), stair climbing score was 2.24±0.82 (range 2-5), and walking activity score was 5.67±2.88 (range 2-5). Mean QF muscle strength was 4.02±0.37 (range 3-5). (Table 2) STS test results found that 33% (n=104) of cases were independent. (Table 2)

**Table 2:** Results of evaluation parameters used in the study.

Degree of knee flexion (°)	100.36±16.45 (35-136)
HSS knee score	58.70±11.08 (22-89)
Pain scores^a^	10.95±7.10 (0-30)
Walking ability^a^	5.67±2.88 (2-5)
Stair climbing ability^a^	2.24±0.82 (2-5)
Transfer ability^a^	2.46±1.09 (2-5)
Functional activity score^a^	10.36±3.69 (4-22)
QF muscle strength	4.02±0.37 (3-5)
STS test	D: 216 (67%) I: 104 (33%)

HSS: Hospital for Special Surgery, D: Dependent, I: Independent, QF: Quadriceps femoris. STS: Sit-to-stand, ^a^Graded by Hospital for Special Surgery score.

Comparison of the relationship between age groups and activity scores found no statistically significant correlation between knee flexion angles, HSS knee score, pain score, scores for walking/stair climbing/transfer, and functional activities scores, QF muscle strength and QF muscle strength scores (p>0.05). (Table 3) Analysis of the relationship between BMI and activity scores revealed a statistically significant difference in terms of HSS knee score, pain score, gait score and functional activity score (p<0.05). (Table 4) HSS knee score, pain score, and walking and functional activity scores were lower in the morbidly obese group (p<0.05). (Table 4)

**Table 3:** Comparison of age groups and activity score.

	40-59 group	60-69 group	70-79 group	80-89 group	f	p
Degree of knee flexion (°)	104.01±16.79	100.53±15.44	98.91±17.27	96.06±13.94	1.72	0.163
HSS knee score	60.87±11.22	57.89±11.99	58.30±10.11	59.20±10.94	1.07	0.360
Pain scores^a^	11.22±6.95	10.30±7.07	10.98±7.21	14.46±6.54	1.58	0.194
Walking ability^a^	6.09±3.07	5.56±3.05	5.59±2.73	5.33±1.95	0.59	0.617
Stair climbing ability^a^	2.19±0.73	2.39±1.01	2.14±0.63	2.20±0.77	2.01	0.112
Transfer ability^a^	2.38±1.00	2.69±1.22	2.37±0.99	2.40±1.05	1.24	0.292
Functional activity score^a^	10.63±3.81	10.58±4.06	10.08±3.38	9.93±2.57	0.55	0.647
QF muscle strength	4.03±0.43	4.05±0.41	4.00±0.29	3.93±0.25	0.60	0.611
QF muscle strength score	7.96±1.21	7.98±1.19	7.93±0.90	7.73±1.03	0.24	0.865

HSS: Hospital for Special Surgery, QF: Quadriceps femoris. ^a^Graded by Hospital for Special Surgery score.

**Table 4:** Comparison of BMI groups and activity score.

	<25 kg/m^2^ group	25-29.9 kg/m^2^ group	30-39.9 kg/m^2^ group	≥40 kg/m^2^ group	f	p
Degree of knee flexion (°)	102.32±13.85	102.15±15.33	99.43±17.13	95.14±19.32	1.465	0.224
HSS knee score	62.82±9.07	61.40±11.73	57.00±10.18	51.14±11.24	8.786	0.000*
Pain scores^a^	13.35±7.89	12.42±7.01	9.87±6.84	7.61±5.61	5.848	0.001*
Walking ability^a^	6.64±2.98	6.02±2.84	5.44±2.80	4.00±2.82	4.663	0.003*
Stair climbing ability^a^	2.44±1.07	2.33±0.95	2.17±0.69	2.00±0.00	2.166	0.092
Transfer ability^a^	2.61±1.23	2.64±1.23	2.36±0.97	2.14±0.65	2.309	0.076
Functional activity score^a^	11.70±4.19	11.00±3.79	9.93±3.42	8.14±2.90	6.100	0.000
QF muscle strength	4.08±0.28	4.06±0.37	3.99±0.38	3.95±0.38	1.405	0.241
QF muscle strength score	8.17±0.57	8.05±1.00	7.86±1.17	7.71±1.30	1.503	0.207

HSS: Hospital for Special Surgery, QF: Quadriceps femoris. ^a^Graded by Hospital for Special Surgery score.

## DISCUSSION

This study investigated the physical and functional characteristics of Turkish patients with OA of the knee. We found that age, sex, and obesity are important factors in the development of OA; walking, stair climbing, transfer and overall functional activity scores are lower in these patients, their knee-joint movements were more limited, and they had high pain scores at rest or during movement.

Reported risk factors for the incidence of knee OA in many countries are obesity (high BMI), sex (female), aging, previous knee trauma, occupational kneeling, squatting, or lifestyle.^1^ ^,^ ^4^ This study also showed that obesity, female sex, and advanced age were significantly associated with an increased risk of radiographic knee OA in Turkish people. The results of our study were consistent with those in the literature.

Age is the greatest risk factor in the development of OA and the prevalence of the disease increases with age, reaching 20% in the 45 years of age group, 40% in the 55 years of age group, 70% in the 65 years of age group, and 80% in geriatric patients over age 75 with osteoarthritis of the knee.^11^ Review of many studies in the literature reveals that the mean age of the OA patients in these studies is 65 years and over.^1^ The mean age of the 320 patients in our study was 66.92 years, which supports the finding that the highest prevalence of osteoarthritis is observed in people aged 60-69. In this study, we found no statistically significant correlation between activity score and the different age groups. (Table 3) Although the functional activity levels of patients with knee OA were seen to decrease due to aging in the literature,^12^ we found no relationship between age and activity levels in the present study. Future studies including more patients could obtain more objective results. Our study also indicates that OA progresses with age, and that patients require more radical surgical treatments in advanced stages of the disease. We consider activities intended to protect the knee (appropriate body weight, adaptive equipment, self-help tools, exercise, recommendations on activities of daily living) useful for healthy aging, and these may also help delay implementation of radical surgeries, such as prosthesis implantation. 

Studies report that the incidence of developing osteoarthritis is higher in females than in males, in different parts of the world.^2^ Women are more prone to knee OA due to several factors, such as changes in QF muscle strength, the presence of less muscle mass and more fat mass, load on joints, pelvic structure, knee morphology, Q angle, neuromuscular strength, hormonal changes occurring with age, and changes in the balance between bone formation and bone resorption.^13^ Women also squat more often than men during daily activities such as going to the toilet and doing housework.^14^ A study conducted in Turkey found that women were expected to perform housework, while men are expected to work outside the home.^15^ The traditional Turkish lifestyle in combination with the decreased muscle strength described above may impact women more than men. Furthermore, older men generally retire from their occupations around 60 to 70 years of age, while women continue to do household chores even after age 70.^7^ All of these reasons may explain why the number of female patients exceeds male patients.^14^ ^-^ ^16^ In line with the literature, of the 320 patients in our study, 257 (80%) were female and 63 (20%) were male. Since Turkish women squat more and are more likely to develop OA, it may be useful to inform them about OA and provide them with preventive physiotherapy.

Obesity is an important but preventable risk factor for osteoarthritis in weight-bearing joints, especially the knee. Studies on this topic found a strong correlation between obesity and knee osteoarthritis.^11,17^ Weight loss can prevent the development of OA and reduce the symptoms of knee OA. The Framingham study found that a weight loss of 5 kg (11 lbs.) in women can reduce the risk of knee osteoarthritis by 50%.^18^ The increasing prevalence of obesity is a significant health problem; there is evidence indicating that obese patients are more likely to require total knee prosthesis (TKP) than non-obese patients. Our study also found a higher proportion of obese patients than non-obese patients. In studies conducted in Turkey, Tekin et al.^16^ found a mean BMI of 33.2 kg/cm^2^ in patients who received TKP; Kocak et al.^3^ found a mean BMI of 30.7 kg/cm^2^ in patients with knee OA (KL 4), and Unver et al.^17^ found a mean BMI of 33.7 kg/cm^2^ in patients who were candidates for TKP. The results of our study agree with these aforementioned data, underscoring the fact that obesity is an important risk factor for OA in Turkish society. The mean age of obese patients was lower than that of non-obese patients, supporting the fact that obesity is a risk factor for knee osteoarthritis and that obese patients require TKP at an earlier age. More than 30% of the Turkish population is obese, suggesting that the prevalence of OA may rise significantly in the future. In the present study, a decrease was observed in patient HSS knee scores, pain scores, and walking and functional activity scores due to increased BMI, in turn leading to a decrease in functional activity levels. (Table 4) Studies have reported that if patients lose weight, reduce existing knee symptoms, and increase their functional activities with a combination of proper diet programs, exercise, weight loss and a combination of lifestyle modifications, they can reduce the rate of overload on the joint due to obesity.^19^ ^,^ ^20^ Therefore, proper diet programs, exercise, and lifestyle changes should be recommended to prevent obesity and ensure that joints remain healthy while aging.^11^

The ability to rise from a chair is an important activity of daily living, and the inability to perform this task may limit independence or lead to institutionalization. The STS test focuses on the knee extensor mechanism and reveals the contraction ability of the QF muscle.^10^ In patients with knee OA, this activity is reduced due to pain and reduced extensor muscle strength. Several studies report that this function is more difficult and takes more time in patients with knee OA compared to healthy subjects.^20^ In our study, 33% of the patients (n=104) did not receive any support while rising from the chair during the STS. We think that this was probably due to fact that our patients with knee OA had sufficient QF muscle strength; we also observed low scores for transfer activity, and that 92% of our study population (n=293) depended on others for these activities.

In patients with knee OA, QF weakness is a clinical feature that has been described several times, and is considered an important determinant of disability.^21^ Knee extensor strength is a highly prevalent and modifiable risk factor for disability in people who have OA and in elderly people without pain.^10^ QF strength is important to maintain dynamic stability during the common basic and instrumental activities of daily living.^10^ Reduced QF muscle strength has been associated with the degree of pain, disability and joint destruction.^22^ However, one study found no correlation between the development of knee OA and QF muscle strength.^2^ Kocak et al.^3^ found a correlation between a higher degree of OA (according to KL) and decreased muscle strength, and reported that improved QF muscle strength would allow patients to perform functional activities of daily living better and be more independent. The study by these authors on QF muscle strength in knee OA patients with varying KL scores found that a muscle strength of 3.7±0.6 in OA patients (KL 4). In this present study, mean QF muscle strength was 4.02±0.37, which was considered good for that KL level, but pain and functional activity levels were found to be low. 

In order to achieve functional activities of daily living, knee flexion of at least 105º is necessary.^23^ The relationship between functional activities and the knee flexion range of motion in patients with knee OA is limited. A study by Kocaket al.^3^ found that as the radiographic grade of OA increases in terms of KL, knee flexion decreases. Our study found mean knee flexion of 100° in our patients with knee OA. This value was not sufficient for these patients to perform activities of daily living. For patients with knee OA to independently perform functional activities of daily living, they should use adaptive practices and self-care tools to facilitate these activities and protect the joint.

## CONCLUSION

This study on Turkish patients with OA determined similar risk factors as the literature. Since obesity is a preventable risk factor, weight loss can prevent the development of OA and reduce symptoms of OA of the knee. Physical inactivity is reported to be one of the most important factors in the development of obesity, so it is very important for patients with knee OA to maintain regular physical activity. It is extremely important to first determine preventable risk factors for OA and then inform patients in order to reduce symptoms after they appear and decrease functional limitations. At this stage, preventive measures may help Turkish patients with knee OA maintain their ideal weights. Moreover, patient education and regular exercise may reduce pain, increasing physical activity and improving quality of life. In this respect, we concluded that in order to boost success in treating knee OA, more objective results can be achieved through studies evaluating risk factors in larger number of patients.
